# Being a relative of a person with cancer can be an emotional burden, but caregiving is often done with love: a survey study amongst relatives

**DOI:** 10.1007/s00520-026-11129-4

**Published:** 2026-08-21

**Authors:** Laurien Ham, Marije van der Lee, Vivian Engelen, Noortje van Willegen

**Affiliations:** 1 Netherlands Comprehensive Cancer Organisation (IKNL), Utrecht, the Netherlands; 2 Helen Dowling Institute, Bilthoven, the Netherlands; 3 CoRPS-Center of Research On Psychological Disorders and Somatic Diseases, Department of Medical and Clinical Psychology, Tilburg University, Tilburg, The Netherlands; 4 Dutch Federation of Cancer Patient Organisations, Utrecht, the Netherlands

**Keywords:** Relatives, Caregiver burden, Cross-sectional, Cancer, Self-reported

## Abstract

**Purpose:**

Understanding how relatives experience the cancer journey and supporting their quality of life is essential to sustain informal care. This study aimed to explore how many relatives experienced a high emotional burden and experienced caregiving as burdensome. Furthermore, we studied the factors associated with their experiences.

**Methods:**

A cross-sectional national exploratory online survey using convenience sampling was conducted among relatives of patients with cancer in the Netherlands. Questions focused on their experiences as a relative, the support they provided and impact on daily life. Univariate logistic regression analyses were performed to identify associated factors.

**Results:**

In total, 3171 relatives completed the survey. The majority (93%) indicated that it feels good to be there for their relative, but most respondents are also afraid of losing their relative to cancer (83%). One in five relatives reported experiencing a significant burden because of being a relative and because of caregiving. Experiencing a high burden was associated with whether a relative fully adjusted their life to that of the patient and whether the relative pretended to be stronger than they felt.

**Conclusion:**

While being a relative of someone with cancer is tough, there are also positive experiences. Caregiving is often done with love, and it can at the same time be an emotional burden. Relatives often pretend to be stronger than they feel. This might aggravate the burden of caregiving. Besides, relatives may benefit from not fully adjusting their lives and taking time for themselves. This may reduce the emotional burden and support their long-term caregiving ability.

**Supplementary Information:**

The online version contains supplementary material available at https://doi.org/10.1007/s00520-026-11129-4.

## Introduction

A cancer diagnosis is a major event for the person diagnosed with cancer as well as for the relatives [1, 2]. Relatives are often involved throughout the cancer journey, from diagnosis and treatment to diverse stages which may include stable disease, recurrence, progression, palliative care and/or end-of-life care [3, 4] and can suffer from feeling helpless and stressed like fear of cancer recurrence/progression as much as the patient. Relatives play a critical role in the overall care of people with cancer; for some relatives, caring for a patient with cancer is equivalent to a full-time job [5, 6]. Hayman et al. reported that patients with cancer received an average of 10 h of informal caregiving per week [7]. Caregiving activities are diverse and numerous, including personal care and management and coordination of medical care, mobility, transportation, communication, housework and emotional support [8, 9]. Many relatives of cancer patients experience a significant burden due to the many responsibilities they carry over a long period of time [2], often without adequate preparation or caregiving skills [10]. Caregiver burden affects, among other things, the mental and physical well-being and social and personal relationships [5, 11–14]. So, the impact of a cancer diagnosis on a relative is twofold: the emotional impact of being a relative of a loved one with cancer and the burden of caregiving.

Several studies show that relatives often fail to address their own needs and feelings while providing support for the patient [15–17]; they put the needs of their loved one before their own needs [18]. Besides the importance of a good quality of life of relatives, the role of the relatives in providing care is becoming increasingly important as the pressure on professional healthcare increases in the Netherlands, with demand for care growing faster than supply [19].

It is important to know how relatives experience the cancer journey and to improve their quality of life so that the necessary informal care can be provided, to the benefit of themselves, the patient, as well as society. Therefore, it is necessary to gain insight into the experience of being a relative and the potential burden associated with it, as well as the burden of providing care. In addition, it is crucial to know what factors are related to the potential burden. Therefore, the aim of this study was to explore how many relatives experienced a high emotional burden of being a relative of someone with cancer and how many experienced *caregiving* as burdensome. Furthermore, we studied the factors associated with their experiences.

## Methods

### Study design

A cross-sectional national exploratory online study was performed in the Netherlands among relatives of cancer patients about their experiences. In this paper, the term ‘relatives’ is used in the broadest sense. It represents partners, family members, friends and all other people personally connected to patients. A questionnaire was initiated and co-created by a patient advocate and a researcher from the Dutch Federation of Cancer Patients Organizations (NFK in Dutch), together with patient representatives of seven cancer patient organizations, experts from the Netherlands Comprehensive Cancer Organization (IKNL) and from the Helen Dowling Institute, an academic mental health institute specialized in cancer related psychological problems. The questionnaire has also been discussed with a communication expert from the Dutch Cancer Association and a representative of MantelzorgNL, a patient organization for informal caregivers. The workgroup met several times to discuss the content of the questionnaire. In between, the workgroup provided (digital) feedback on draft versions of the questionnaire.

### Eligibility criteria

The questionnaire was open to all (bereaved) relatives of cancer patients. The first question was an explanation of the target group for the survey and the question ‘Do you or did you have a close other with cancer?.’ If this was answered positively, then the rest of the questions appeared. If it was answered negatively, the respondent was excluded from further participation.

### Data collection

Between December 2022 and January 2023, the NFK distributed the questionnaire for 6 weeks through their website ‘www.doneerjeervaring.nl’ various social media (Facebook, Instagram, LinkedIn and Twitter), and among their network of (former) cancer patients and their relatives. Cancer patient organizations—united within NFK—sent their constituencies (member or donors) an e-mail inviting them to complete the questionnaire. They also used various social media to recruit respondents. In addition, members of the ‘Donate Your Experience’ (DJE) panel received an e-mail invitation for the questionnaire. Besides, also other Dutch cancer organizations like the Dutch Cancer Association and some hospitals have asked their contacts to fill out the questionnaire. The questionnaire was anonymous and the average completion time was 15 min.

The background information collected that was most relevant for this paper: relation to the patient, gender, age group (now and at the moment of diagnosis), educational status, living situation, disease status and cancer type. The questions mainly concerned the experience of being a relative of a person with cancer, the support they gave and how they experienced giving that support and the consequences for their daily live. All questions were closed-ended (multiple choice or Likert scales), including, where relevant, an ‘other’ category, except for three questions (‘What helps or helped you the most as a relative of someone with cancer?’; ‘As a relative of someone with cancer, what do you miss or missed the most?’ and ‘Do you have tips for other relatives of people with cancer?’) (for an overview of answers to the questions by type of cancer, age and type of relationship, see Supplementary table S1).

### Participants

Participants were informed about privacy regulations, in accordance with the General Data Protection Regulation (EU). The Medical Research Involving Human Subjects Act (WMO) did not apply since the study did not include an intervention wherefore ethical approval was not needed. Consent was provided by all participants who anonymously filled in the online questionnaire.

### Data analyses

To answer the research question of this paper, in-depth analyses were performed on the collected data. No minimum sample size was calculated prior to the study since this study was explorative in nature. Descriptive statistics about the emotional burden of being a relative and experiences with caregiving to patients were reported per age category (either younger or older than 60 years), per cancer type, rare or common cancer type and per type of relationship with the person with cancer. Absolute numbers and percentages were provided for nominal variables. Means and standard deviations were provided for continuous variables. We calculated two variables that summarize the emotional burden of being a relative and the experienced burden of caregiving. Analyses were performed using available-case (complete-case) data for each individual analysis. As a result, denominators may vary slightly across items depending on the number of missing responses.

Emotional burden of being a relative was defined as the emotional and psychological strain associated with having a close other with cancer, reflected in feelings of helplessness, tiredness, loneliness, insufficient self-care and not feeling understood by others. We operationalized **emotional burden of being a relative** as high when 4 out of the following 5 questions were agreed upon (either agree or totally agree was answered):

As a relative of a patient with cancer;

1. I feel helpless
2. I feel tired
3. I feel lonely
4. I don’t take good care of myself
5. I don’t feel understood by those around me

Experiencing burden of caregiving was defined as the perceived strain associated with providing support or help to a close other with cancer, reflected in emotional and physical heaviness, limited social life due to caregiving and difficulty combining caregiving with other daily responsibilities. We operationalized **experiencing caregiving as burdensome** as: when 3 out of the following 4 questions were agreed upon (either agree, or totally agree was answered):

1. Caregiving is emotionally heavy
2. Caregiving is physically heavy
3. Due to caregiving I have no social life
4. It is too much for me to give support or help, besides all other tasks of daily life

Logistical univariate regression analyses were performed to identify which factors were associated with experiencing emotional burden and with experiencing caregiving as burdensome as outcomes. The following demographic factors were included in this analysis: age at diagnosis (dichotomized), rare tumour type or not, disease phase, type of relationship with the patient (i.e., being a partner was the reference group and compared with parent, child, friend, sibling and other relationship), gender and education (practical, middle and high; included as ordinal variable in the regression analysis). The following behavioural factors were included: I completely adapt my life to that of my relative with cancer, as a relative of someone with cancer I pretend to be stronger than I am, I am grateful to be able to give care. The last behavioural factor was only answered by relatives who reported caring for the patient. IBM SPSS Statistics version 28 was used for all analyses.

## Results

In total, 4652 respondents started the online survey. Of these participants, 71 (2%) did not meet the inclusion criteria (i.e., they had or have no relative with cancer) and 1410 (30%) did not complete the questionnaire. After these exclusions, 3171 (68%) relatives were eligible for analysis. 75% of the relatives were female, and 51% of the relatives were 60 years or older when completing the questionnaire. More than half of the respondents (59%) were the partner of the patient with cancer. 33% of the relatives indicated that the patient was (probably) cured from cancer, 30% indicated that the patient was (probably) not cured and 33% of the relatives indicated that the patient has died. Then, 45% were a relative of a patient with a rare cancer, 13% were relatives of patients with prostate cancer and 11% of patients with breast cancer (Table 1).

**Table 1:** Sociodemographic characteristics of relatives (*n* = 3171)

	***N***	**%**
Female	2374	75%
*Age at completing questionnaire*		
< 60 years old	1556	49%
≥ 60 years old	1615	51%
*Age at cancer diagnosis*		
< 60 years old	1644	52%
≥ 60 years old	1525	48%
*Type of relationship with the patient*		
Partner	1876	59%
Child	504	16%
Parent	279	9%
Sibling	241	8%
Friend	143	5%
Other family	98	3%
Other	30	1%
*Rare vs. common cancer*		
Rare cancer	1412	45%
Common cancer	1721	54%
Unknown	38	1%
*Tumour type (5 most reported)*		
Breast cancer	347	11%
Prostate cancer	419	13%
Lung cancer	314	10%
Pancreas cancer	139	4%
Colon cancer	193	6%
*Cancer stage*		
(Probably) cured	1054	33%
(Probably) not cured	944	30%
Deceased	1051	33%
Unknown	122	4%
*Educational level*		
Practical	481	15%
Middle	1148	36%
High	1464	46%

### Emotional burden of being a relative of a patient with cancer

**Fig. 1:**
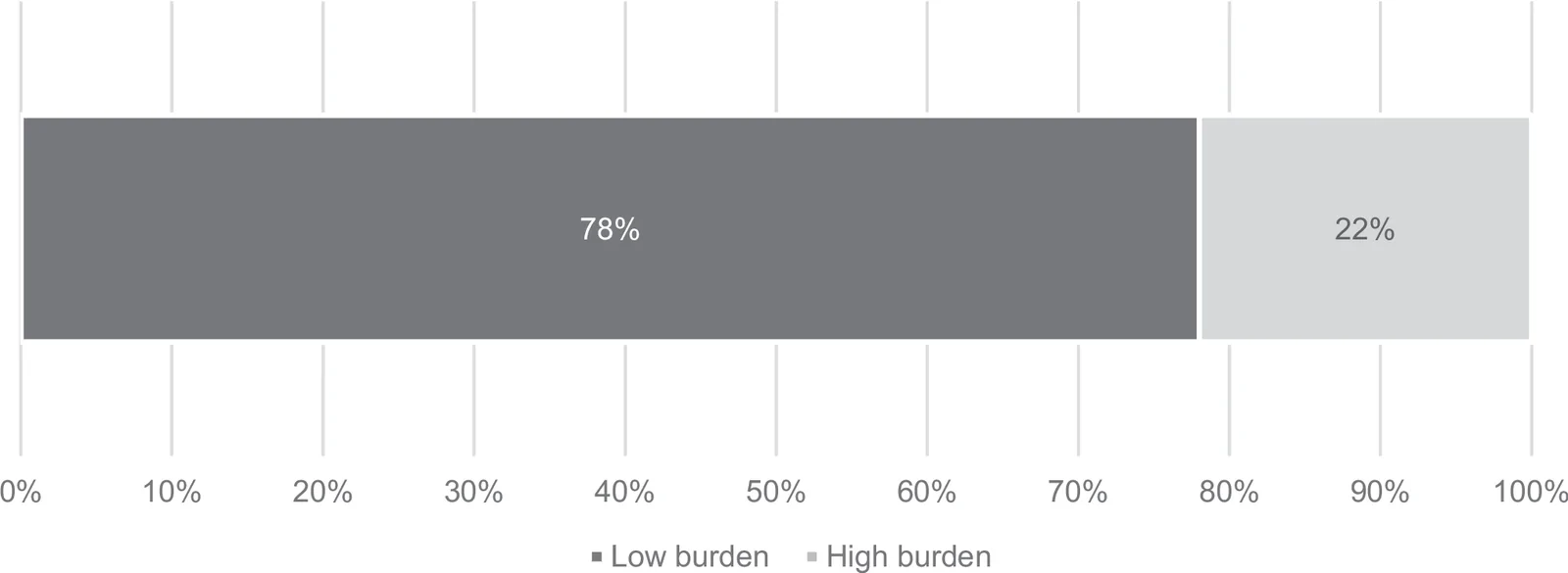
Number of relatives (%) who experienced emotional burden of being a relative of a patient with cancer (*N* = 3171)

Almost all respondents indicated that it feels good to be able to be there for their relative (93%), but most respondents also indicated that they are afraid of losing their relative to cancer (83%) and feel helpless as relative of someone with cancer (73%). Two-thirds of the respondents (67%) pretended to be stronger than they were, and half of the respondents (55%) adapted their own lives completely to that of their relative with cancer (Table 2). Over 20% of the respondents experienced being a relative of someone with cancer as a high emotional burden (Fig. 1). A selection of responses to the qualitative questions can be found in Supplementary Table S2.

**Table 2:** Number of relatives who (totally) agree with the statements about the experienced emotional burden of being a relative of a patient with cancer (*N* = 3171)

	(Totally) agree (%)
It feels good to be able to be there for my relative with cancer	93%
I am afraid of losing my relative with cancer	83%
I feel helpless as a relative of someone with cancer	73%
As a relative of someone with cancer, I hold myself stronger than I am	67%
I completely adapt my own life to that of my relative with cancer	55%
I feel strong as a relative of someone with cancer	50%
I feel tired as a relative of someone with cancer	48%
The relationship with my relative improved greatly after he/she got cancer	42%
I feel lonely as a relative of someone with cancer	39%
The relationship with my family improved greatly after my relative got cancer	29%
As a relative of someone with cancer, I don’t take good care of myself	26%
I don't feel understood by those around me as a relative of someone with cancer	25%
Because my relative got cancer, I am afraid of getting getting cancer myself	25%

Univariate analyses revealed that experiencing higher emotional burden of being a relative was significantly associated with type of relationship diagnosis (children have a slightly higher risk and siblings and others a lower risk compared to the reference group of partners), lower age (being under 60 at diagnosis) of the relative at diagnosis (OR = 0.66, [95% CI 0.60–0.73]), a rare cancer diagnosis in comparison with a common cancer diagnosis (OR = 1.26, [95% CI 1.07–1.50]), a higher education level (OR = 1.15, [95% CI 1.02–1.30]), being a relative of a patient who was (likely to be) not cured or deceased (OR = 1.40, [95% CI 1.16–1.68]), female gender (OR = 1.85, [95% CI 1.48–2.30]), the relative adjusted their life completely to that of the patient with cancer (OR = 4.42, [95% CI 3.60–5.44]) and the relative pretended to be stronger than he/she is (OR = 32.45, [95% CI 22.97–45.85]) (Table 3).

**Table 3:** Association between the risk factors and experiencing emotional burden of being a relative of a patient with cancer

	Odds ratio	*p*-value	95% confidence interval
Age at diagnosis			
< 60 years old	Ref		
> 60 years old	0.66	< 0.001	[0.60–0.73]
Type of relationship			
Partner	Ref		
Child	1.28	0.03	[1.02–1.60]
Parent	1.26	0.11	[0.95–1.68]
Friend	0.42	0.002	[0.24–0.72]
Sibling	0.73	0.08	[0.51–1.04]
Other	0.32	0.002	[0.15–0.66]
Type of cancer			
Rare cancer	Ref		
Common cancer	1.26	0.01	[1.07–1.50]
Education level (ordinal variable: practical-middle-high)	1.15	0.02	[1.02–1.30]
Stage of cancer			
(likely to be) cured	Ref		
(likely to be) incurable or deceased	1.40	< 0.001	[1.16–1.68]
Gender			
Male	Ref		
Female	1.85	< 0.001	[1.48–2.30]
I completely adjust my own life to that of my relative with cancer			
No	Ref		
Yes	4.42	< 0.001	[3.60–5.44]
As a relative of someone with cancer, I hold myself stronger than I am			
No	Ref		
Yes	32.45	< 0.001	[22.97–45.85]
I am grateful that I can give support or help			
No	Ref		
Yes	0.92	0.54	[0.70–1.21]

Analyses were performed using available-case (complete-case) data for each individual analysis. As a result, *N* may vary slightly across items depending on the number of missing responses

### Experienced burden of caregiving

**Fig. 2:**
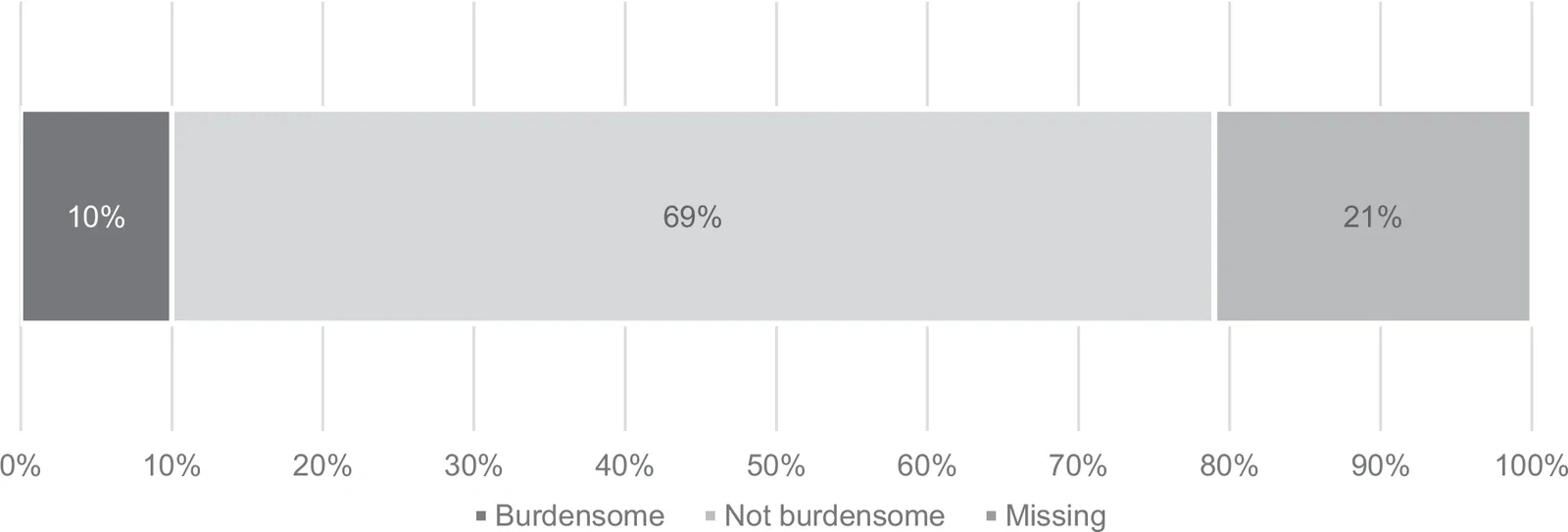
Number of relatives (%) who experienced caregiving as burdensome (*N* = 3171)

In addition, 10% of the respondents experienced caregiving as burdensome (Fig. 2). Univariate analyses revealed that experiencing caregiving as burdensome was significantly associated with type of relationship (with being partners associated with higher burden compared to siblings); a lower education level (OR = 0.79, [95% CI 0.67–0.93]), being a relative of a patient who was (likely to be) incurable or deceased (OR = 1.86, [95% CI 1.41–2.45]), female gender (OR = 1.82, [95% CI 1.33–2.49]), the relative adjusted their life completely to that of the patient with cancer (OR = 7.56, [95% CI 5.20–11.00]) and the relative pretended to be stronger than he/she feels (OR = 11.07, [95% CI 7.57–16.20]). (Table 4).

**Table 4:** Association between the risk factors and experiencing giving support as hard

	Odds ratio	*p*-value	95% confidence interval
Age at diagnosis			
< 60 years old	Ref		
> 60 years old	0.89	0.13	[0.77–1.03]
Type of relationship			
Partner	Ref		
Child	1.10	0.57	[0,80–1,51]
Parent	1.11	0.60	[0,74–1,68]
Friend	0.18	0.004	[0,06–0,58]
Sibling	0.66	0.13	[0,39–1,13]
Other	0.99	0.99	[0,49–2,05]
Type of cancer			
Rare cancer	Ref		
Common cancer	1.27	0.05	[0.997–1.60]
Education level (ordinal variable: practical-middle-high)	0.79	0.01	[0.67–0.93]
Stage of cancer			
(likely to be) cured	Ref		
(likely to be) incurable or deceased	1.86	< 0.001	[1.41–2.45]
Gender			
Male	Ref		
Female	1.82	< 0.001	[1.33–2.49]
I completely adapt my own life to that of my relative with cancer			
No	Ref		
Yes	7.56	< 0.001	[5.20–11.00]
As a relative of someone with cancer, I hold myself stronger than I am			
No	Ref		
Yes	11.07	< 0.001	[7.57–16.20]
I am grateful that I can give support or help			
No	Ref		
Yes	0.95	0.79	[0.66–1.37]

Analyses were performed using available-case (complete-case) data for each individual analysis. As a result, *N* may vary slightly across items depending on the number of missing responses

## Discussion

In the survey, almost all 3171 relatives said that it felt good to be able to be there for the patient, and at the same time, they were afraid of losing their relative to cancer and felt helpless. Two-thirds of the respondents masked their true feelings by pretending to be stronger than they felt, and half of the respondents completely altered their lives to accommodate the needs of their loved ones. One in five relatives reported experiencing a significant burden being a relative: “The world gets smaller, more intimate, the time we were allowed to receive, we enjoyed that more consciously with sadness, helplessness but also joy and gladness for small and big blessings”. Experiencing a high emotional burden of being a relative was associated with several factors including being younger at time of diagnosis. Most notably, both emotional burden of being a relative and experiencing caregiving as burdensome were closely tied to whether a relative fully adjusted their life completely to that of the patient and whether the relative pretended to be stronger than they felt.

It is known from the literature that relatives experience many problems and increased responsibilities during and after the patient is undergoing treatment and rehabilitation for cancer [2]. However, our study showed that relatives also experience positive consequences of being there for the patient. This was also found in a qualitative study among relatives, which revealed that some relatives saw this as a chance to give something back to the patient [20]. Other studies revealed that relatives perceive their experiences as informal caregiver as positive [21] and relatives report feelings of participation in a meaningful experience [22] a strengthened relationship [23–26] and personal growth [23, 26].

Our study revealed that a higher level of education was associated with a higher emotional burden of being a relative, but experiencing caregiving as more burdensome was associated with a lower level of education. These contrasting results are also seen in the literature. Most studies in the cancer literature show that a higher education level was associated with better coping mechanisms, better mental adjustment, lower stress levels and better quality of life [27, 28]. However, other studies found higher educational level to be associated with higher emotional stress and a higher burden [29, 30]. Moreover, in studies among relatives of patients with dementia, higher educational level has more often been positively associated with caregiver burden [31]. This may be explained by a better understanding of the complexity and consequences of the disease, different expectations regarding treatment or caregiving [31] and a sense of duty or social obligation to care for relatives [29].

We found that being a woman was associated with a higher burden. This is in line with the literature [32]. The differences between men and women can be explained by differences in the levels of stress and coping strategies employed by men and women. Women experience higher levels of stress and their coping style is more emotional-focused [33, 34].

Pretending to be stronger than someone actually is may be a coping strategy to deal with the situation, although people are also told by others that they must stay strong as demonstrated by some quotes. It may help on the short term to deal with the situation, but as our study revealed, it also makes it more burdensome. To the best of our knowledge, little research has been done on pretending to be stronger than you are in the context of cancer. However, a review has been done in the context of *the strong black woman* construct in relation to health [35]. This review found that portrayal of strength and resilience was related to several health problems (such as hypertension and heart disease) and depression and anxiety symptoms [35].

### Study limitations

This study has several limitations. Firstly, it is a survey study in which a convenience sample of respondents answered questions about their experience that were sometimes years ago. This may have led to some hindsight bias, although in general the results of the survey revealed both positive as well as negative experiences. Secondly convenience sampling may lead to selection bias, so the respondents may not be representative for all relatives; three-quarters were female and almost half of the respondents were highly educated. With respect to cancer types, all cancer types were represented. Thirdly, the questionnaire was only available in Dutch, which prevented non-Dutch-speaking residents from completing the questionnaire. Fourthly, a nonvalidated questionnaire was used and the thresholds chosen to identify burden were only based on face validity. Furthermore, the strongest associations found were with variables that reflect behavioural responses related to burden namely adapting one’s life and pretending to be stronger, which may have some conceptual overlap. Fifthly, since the results were cross-sectional, we cannot draw any causal conclusions based on the data. Finally, the questionnaire was probably mainly filled in by relatives that are connected to patient organizations, which might not be representative of all cancer patients; therefore, we emphasize that findings should be interpreted with caution.

### Clinical implication

The most important associations with the perceived difficulty of providing support were that relatives had adjusted their lives completely to align with that of the cancer patient, and they often felt compelled to appear stronger than they truly were. It is always difficult to predict the course of cancer, especially when the prognosis is uncertain. This can lead to a long and uncertain trajectory. The advice for relatives would be to not completely adjust their life to the patient with cancer, because they may be needed for a long time and it is important to have some time for themselves in order to stay healthy enough, also to be able to provide care. Pretending to be stronger may be done to protect the patient, but it is important that relatives know this might come with an extra risk for burden. They might be stimulated to seek social support with whom they can share that they do not always feel that strong. Online social support groups are also known to provide emotional support to relatives, which might be a good solution when relatives are short of time [36]. Also, they can consult their general practitioner, or the patient’s doctor or nurse. However, since we saw that most relatives pretended to be stronger than they actually were, it is to be expected that those relatives will not ask for help for themselves. Therefore, there is also an important role for the healthcare professionals, patient organizations and friends and family of the relative in recognizing when a relative needs help and to give them support and advice.

## Conclusion

Being a relative of someone with cancer is tough, but there are also positive experiences, as said by someone “The worst time, but also the most beautiful time”. Caregiving is often done with love, and it can at the same time be an emotional burden. Relatives often pretend to be stronger than they feel. This might aggravate the burden of caregiving.

## Supplementary Information

Below is the link to the electronic supplementary material.

ESM 1 (DOCX 32.4 KB)

EESM 2 (DOCX 21.5 KB)

## Data Availability

Data are available from and with permission from the Dutch Federation of Cancer Patient Organizations (NFK).
