# Supplementary material for: Being a relative of a person with cancer can be an emotional burden, but caregiving is often done with love: a survey study amongst relatives
Source: Support Care Cancer. 2026 Aug 21;34(9):884. doi: 10.1007/s00520-026-11129-4 (PMC13498499; doi:10.1007/s00520-026-11129-4)
Supplement: Supplementary file 1 [file 520_2026_11129_MOESM1_ESM.docx]

Supplementary table S1: experiences of relatives of patients with cancer, by type of cancer, age and type of relationship (N = 3171)

|  | **Type of cancer**  **N that agreed (%)** | | **Age at cancer diagnosis of relative**  **N that agreed (%)** | | | | | **Type of relationship with the patient**  **N that agreed (%)** | | | | | |
| --- | --- | --- | --- | --- | --- | --- | --- | --- | --- | --- | --- | --- | --- |
|  | *Common cancer* | *Rare cancer* | *≤17 years old* | *18 - 39 years old* | *40-59 years old* | *60-74 years old* | *≥75 years old* | *Spouse* | *Child* | *Parent* | *Friend* | *Sibling* | *Other* |
| I like being there for my relative with cancer | 1610 (94%) | 1320 (94%) | 77  (91%) | 518 (96%) | 1346 (94%) | 934 (94%) | 92  (94%) | 1767 (95%) | 475 (95%) | 257 (94%) | 131 (92%) | 221 (93%) | 90  (92%) |
| I completely adapt my own life to that of my relative with cancer | 861 (51%) | 851 (62%) | 36  (46%) | 278 (52%) | 826 (58%) | 527 (54%) | 65  (67%) | 1151 (62%) | 250 (51%) | 175 (65%) | 28  (20%) | 91  (39%) | 26  (29%) |
| The relationship with my family improved greatly after my relative got cancer | 443 (27%) | 440 (32%) | 26  (31%) | 173 (33%) | 399 (29%) | 263 (28%) | 33  (34%) | 456 (25%) | 183 (37%) | 98  (37%) | 13  (11%) | 104 (45%) | 34  (36%) |
| The relationship with my relative improved greatly after he/she got cancer | 725 (44%) | 614 (46%) | 33  (42%) | 248 (49%) | 633 (45%) | 399 (42%) | 37  (38%) | 749 (41%) | 210 (44%) | 131 (52%) | 76  (56%) | 135 (59%) | 38  (44%) |
| I feel strong as a relative of someone with cancer | 875 (52%) | 697 (50%) | 24  (28%) | 201 (38%) | 689 (48%) | 611 (62%) | 64  (63%) | 1073 (58% | 192 (39%) | 140 (52%) | 51  (36%) | 95  (40%) | 27  (28%) |
| I feel lonely as a relative of someone with cancer | 622 (37%) | 601 (43%) | 39  (46%) | 258 (48%) | 618 (44%) | 297 (30%) | 28  (28%) | 742 (40%) | 217 (44%) | 125 (46%) | 42  (30%) | 90  (38%) | 16  (18%) |
| I don't feel understood by those around me as a relative of someone with cancer | 393 (23%) | 383 (28%) | 37  (44%) | 213 (40%) | 368 (26%) | 157 (16%) | 13  (13%) | 447 (24%) | 162 (33%) | 80  (30%) | 28  (20%) | 51  (22%) | 9  (9%) |
| I feel powerless as a relative of someone with cancer | 1245 (73%) | 1059 (75%) | 72  (83%) | 458 (85%) | 1108 (77%) | 636 (64%) | 58  (58%) | 1279 (69%) | 429 (85%) | 240 (87%) | 104 (73%) | 189 (80%) | 73  (75%) |
| I feel tired as a relative of someone with cancer | 752 (45%) | 750 (54%) | 25  (31%) | 293 (55%) | 773 (54%) | 393 (40%) | 38  (39%) | 934 (50%) | 258 (52%) | 166 (61%) | 32  (23%) | 92  (40%) | 29  (31%) |
| I am afraid of losing my relative with cancer | 149 (84%) | 1193 (87%) | 73  (87%) | 478 (90%) | 1224 (87%) | 789 (81%) | 69  (73%) | 1542 (84%) | 427 (88%) | 245 (91%) | 116 (82%) | 202 (89%) | 78  (82%) |
| As a relative of someone with cancer, I hold myself stronger than I am | 1099 (64%) | 986 (70%) | 63  (73%) | 425 (79%) | 983 (69%) | 585 (59%) | 56  (56%) | 1210 (65%) | 372 (74%) | 221 (81%) | 83  (58%) | 155 (65%) | 55  (57%) |
| As a relative of someone with cancer, I take poor care of  myself | 406 (24%) | 383 (27%) | 26  (31%) | 183 (34%) | 403 (28%) | 177 (18%) | 14  (14%) | 467 (25%) | 165 (33%) | 87  (32%) | 22  (16%) | 45  (19%) | 10  (11%) |
| Because my relative got cancer, I am afraid of getting  getting cancer myself | 448 (27%) | 337 (25%) | 48  (56%) | 236 (44%) | 361 (26%) | 141 (15%) | 5  (5%) | 302 (17%) | 239 (48%) | 81  (30%) | 45  (33%) | 89  (39%) | 27  (28%) |
