# Supplementary material for: Being a relative of a person with cancer can be an emotional burden, but caregiving is often done with love: a survey study amongst relatives
Source: Support Care Cancer. 2026 Aug 21;34(9):884. doi: 10.1007/s00520-026-11129-4 (PMC13498499; doi:10.1007/s00520-026-11129-4)
Supplement: Supplementary file 2 [file 520_2026_11129_MOESM2_ESM.docx]

Supplementary table S2: Quotes of relatives illustrating the experiences of being a relative of a patient with cancer and the experiences with giving support

**Quotes illustrating the experienced burden of being a relative and the experienced burden of caregiving**

“The worst time, but also the most beautiful time”

“Everything revolves around the partner, hospital visits, dealing with the examination results, informing the children, family, etc. Dealing with reactions of others, keeping things running; emotionally and physically a huge effort. Done with love, but it was tough.”

“I did it with love. I also knew he only had a short time to live and wanted to take care of him with heart of soul. I felt powerful. Yet I also undertook my own activities in order to recharge and not be constantly on my husband’s back”

“I take it for granted that I give help, but I notice that emotionally it gets heavier and heavier. All the attention goes to the patient and the relative is often forgotten.”

“Everything changed. In the beginning I was tiptoeing more, not daring to go (far) away, not daring to bicker. It’s wave movements. Sometimes it overwhelms you, sometimes you seem to have forgotten it for a moment. But somewhere it is always there; sometimes in the background, sometimes right in front of you. I am afraid of what is to come.”

"The world gets smaller, more intimate the time we were allowed to receive, we enjoyed that more consciously with sadness, helplessness but also joy and gladness for small and big blessings."

"Because we are quite young (both 30 at diagnosis) I was really pushed aside by the family in the beginning (even though we had been together for 10 years and living together for 6)."

"Taking care of your partner, being there for that one. I did it with a lot of love. You are together in good times and bad. It was tough and sometimes difficult. But it was also nice to be able to do it. Together with the children."

“What I especially had was that it was very exhausting mentally, because my husband and I both had to deal with it in a very different way for us personally (this exhausted me mentally tremendously) and that you don't feel yourself seen as a partner anymore during the process."

“How to share your own emotions, as well as those of your partner, without emotionally overwhelming him, and without always feeling the need to be strong. This became a heavy burden on me, especially after the sudden passing of my partner.”
